# Transcriptomic profile of cationic channels in human pulmonary arterial hypertension

**DOI:** 10.1038/s41598-021-95196-z

**Published:** 2021-08-04

**Authors:** Francisco Perez-Vizcaino, Angel Cogolludo, Gema Mondejar-Parreño

**Affiliations:** 1 Department of Pharmacology and Toxicology. School of Medicine, Universidad Complutense de Madrid, Madrid, Spain; 2 Ciber Enfermedades Respiratorias (Ciberes), Madrid, Spain; 3 Instituto de Investigación Sanitaria Gregorio Marañón (IISGM), Madrid, Spain

**Keywords:** Molecular biology, Molecular medicine

## Abstract

The dysregulation of K^+^ channels is a hallmark of pulmonary arterial hypertension (PAH). Herein, the channelome was analyzed in lungs of patients with PAH in a public transcriptomic database. Sixty six (46%) mRNA encoding cationic channels were dysregulated in PAH with most of them downregulated (83%). The principal component analysis indicated that dysregulated cationic channel expression is a signature of the disease. Changes were very similar in idiopathic, connective tissue disease and congenital heart disease associated PAH. This analysis 1) is in agreement with the widely recognized pathophysiological role of TASK1 and K_V_1.5, 2) supports previous preliminary reports pointing to the dysregulation of several K^+^ channels including the downregulation of K_V_1.1, K_V_1.4, K_V_1.6, K_V_7.1, K_V_7.4, K_V_9.3 and TWIK2 and the upregulation of K_Ca_1.1 and 3) points to other cationic channels dysregulated such as Kv7.3, TALK2, Ca_V_1 and TRPV4 which might play a pathophysiological role in PAH. The significance of other changes found in Na^+^ and TRP channels remains to be investigated.

## Introduction

Pulmonary arterial hypertension (PAH) is a severe disease characterized by an abnormally high mean pulmonary arterial pressure due to distal pulmonary vessel structural remodeling, altered pulmonary arterial tone and inflammation, causing right ventricular hypertrophy and right heart failure^1,2^. Some characteristic pathophysiological features of the disease include an imbalance between the TGF-β/BMPR2 pathways, endothelial dysfunction, mitochondrial dysfunction and dysregulation of several ion channels, leading to vasoconstriction, changes in vascular cell phenotype, proliferation, de-differentiation and inflammation^2^. The disease causes a significant burden in terms of quality of life and entails a poor prognosis.

Cationic channels include K^+^, Na^+^, Ca^2+^ and non-selective cationic channels^3^. The K^+^ channels superfamily comprises 4 main families: voltage-gated (K_V_), inward rectifier (K_IR_), Ca^2+^-activated (K_Ca_) and two-pore-domain (K_2P_) K^+^ channels. Other cationic channel families include voltage-dependent Na^+^ and Ca^2+^ channels (Na_V_ and Ca_V_), transient receptor potential channels (TRP) and cyclic nucleotide gated channels (CNG and HCN). All these families have evolved from a common ancestor, share structural similarities and have been classified together as the "voltage-gated-like” ion channels^3^. The families, subfamilies, gene names and the corresponding encoded proteins of the channels studied herein are shown in Table 1.

**Table 1:** Ion channel encoding genes and the corresponding cationic channel proteins.

Fam	Subfam	Gene	Channel	Fam	Subfam	Gene	Channel	Fam	Subfam	Gene	Channel
K_V_	K_V_1.x	*KCNA1*	K_V_1.1	K_2P_	K2P	*KCNK1*	TWIK	TRP	TRPA	*TRPA1*	TRPA1
		*KCNA2*	K_V_1.2			*KCNK2*	TREK1		TRPC	*TRPC1*	TRPC1
		*KCNA3*	K_V_1.3			*KCNK3*	TASK1			*TRPC2*	TRPC2
		*KCNA4*	K_V_1.4			*KCNK4*	TRAAK1			*TRPC3*	TRPC3
		*KCNA5*	K_V_1.5			*KCNK5*	TASK2			*TRPC4*	TRPC4
		*KCNA6*	K_V_1.6			*KCNK6*	TWIK2			*TRPC5*	TRPC5
		*KCNA7*	K_V_1.7			*KCNK7*	K2P7.1			*TRPC6*	TRPC6
		*KCNA10*	K_V_1.8			*KCNK9*	TASK3			*TRPC7*	TRPC7
	K_V_2.x	*KCNB1*	K_V_2.1			*KCNK10*	TREK2		TRPM	*TRPM1*	TRPM1
		*KCNB2*	K_V_2.2			*KCNK12*	THIK2			*TRPM2*	TRPM2
	K_V_3.x	*KCNC1*	K_V_3.1			*KCNK13*	THIK1			*TRPM3*	TRPM3
		*KCNC2*	K_V_3.2			*KCNK15*	TASK5			*TRPM4*	TRPM4
		*KCNC3*	K_V_3.3			*KCNK16*	TALK1			*TRPM5*	TRPM5
		*KCNC4*	K_V_3.4			*KCNK17*	TALK2			*TRPM6*	TRPM6
	K_V_4.x	*KCND1*	K_V_4.1			*KCNK18*	TRESK			*TRPM7*	TRPM7
		*KCND2*	K_V_4.2							*TRPM8*	TRPM8
		*KCND3*	K_V_4.3	K_Ca_	KCa	*KCNMA1*	KCa1.1		TRPV	*TRPV1*	TRPV1
	K_V_5.x	*KCNF1*	K_V_5.1			*KCNN1*	KCa2.1			*TRPV2*	TRPV2
	K_V_6.x	*KCNG1*	K_V_6.1			*KCNN2*	KCa2.2			*TRPV3*	TRPV3
		*KCNG2*	K_V_6.2			*KCNN3*	KCa2.3			*TRPV4*	TRPV4
		*KCNG3*	K_V_6.3			*KCNN4*	KCa3.1			*TRPV5*	TRPV5
		*KCNG4*	K_V_6.4		KNa	*KCNT1*	KNa1.1			*TRPV6*	TRPV6
	K_V_7.x	*KCNQ1*	K_V_7.1			*KCNT2*	KNa1.2		TRPML	*MCOLN1*	TRPML1
		*KCNQ2*	K_V_7.2							*MCOLN2*	TRPML2
		*KCNQ3*	K_V_7.3	Ca_V_	Ca_V_1.x	*CACNA1S*	Ca_V_1.1			*MCOLN3*	TRPML3
		*KCNQ4*	K_V_7.4			*CACNA1C*	Ca_V_1.2		TRPP	*PKD2*	TRPP1
		*KCNQ5*	K_V_7.5			*CACNA1D*	Ca_V_1.3			*PKD2L1*	TRPP2
	K_V_8.x	*KCNV1*	K_V_8.1			*CACNA1F*	Ca_V_1.4			*PKD2L2*	TRPP3
		*KCNV2*	K_V_8.2		Ca_V_2.x	*CACNA1A*	Ca_V_2.1		TPCN	*TPCN1*	TPCN1
	K_V_9.x	*KCNS1*	K_V_9.1			*CACNA1B*	Ca_V_2.2			*TPCN2*	TPCN2
		*KCNS2*	K_V_9.2		Ca_V_3.x	*CACNA1E*	Ca_V_2.3				
		*KCNS3*	K_V_9.3			*CACNA1G*	Ca_V_3.1	CNG HCN	CNGA	*CNGA1*	CNGA1
	K_V_10	*KCNH1*	K_V_10.1			*CACNA1H*	Ca_V_3.2			*CNGA2*	CNGA2
		*KCNH5*	K_V_10.2			*CACNA1I*	Ca_V_3.3			*CNGA3*	CNGA3
	K_V_11.x	*KCNH2*	K_V_11.1							*CNGA4*	CNGA4
		*KCNH6*	K_V_11.2	ORAI	ORAI	*ORAI1*	ORAI1		CNGB	*CNGB1*	CNGB1
		*KCNH7*	K_V_11.3			*ORAI2*	ORAI2			*CNGB3*	CNGB3
	K_V_12.x	*KCNH8*	K_V_12.1			*ORAI3*	ORAI3		HCN	*HCN1*	HCN1
		*KCNH3*	K_V_12.2							*HCN2*	HCN2
		*KCNH4*	K_V_12.3	Na_V_	Na_V_1.x	*SCN1A*	Na_V_1.1			*HCN3*	HCN3
						*SCN2A*	Na_V_1.2			*HCN4*	HCN4
K_IR_	K_IR_1.x	*KCNJ1*	K_IR_1.1			*SCN3A*	Na_V_1.3				
	K_IR_2.x	*KCNJ2*	K_IR_2.1			*SCN4A*	Na_V_1.4				
		*KCNJ12*	K_IR_2.2			*SCN5A*	Na_V_1.5				
		*KCNJ4*	K_IR_2.3			*SCN8A*	Na_V_1.6				
		*KCNJ14*	K_IR_2.4			*SCN9A*	Na_V_1.7				
	K_IR_3.x	*KCNJ3*	K_IR_3.1			*SCN10A*	Na_V_1.8				
		*KCNJ6*	K_IR_3.2			*SCN11A*	Na_V_1.9				
		*KCNJ9*	K_IR_3.3		Na_V_2.x	*SCN7A*	Na_V_2.1				
		*KCNJ5*	K_IR_3.4								
	K_IR_4.x	*KCNJ10*	K_IR_4.1	Na_VI_	Na_VI_2.x	*NALCN*	Navi2.1				
		*KCNJ15*	K_IR_4.2								
	K_IR_5.x	*KCNJ16*	K_IR_5.1								
	K_IR_6.x	*KCNJ8*	K_IR_6.1								
		*KCNJ11*	K_IR_6.2								
	K_IR_7.x	*KCNJ13*	K_IR_7.1								

A wide variety of cationic channels are functionally expressed in pulmonary artery smooth muscle (PASMC) and endothelial (PAEC) cells^4–6^. K^+^ channel conductance plays a fundamental role in the control of their membrane potential (Em), which regulates the cell excitability and the activity of other voltage-gated channels including Ca^2+^ channels^6^. Ca^2+^ conductance in the cell membrane is the main determinant of cytosolic Ca^2+^ which in turn regulates the contractility of pulmonary arteries, the release of endothelial factors and neurotransmitters and the cell proliferation/apoptotic balance. Therefore, an adequate vascular K^+^ and Ca^2+^ channel function ensures an optimal blood flow distribution within the lungs, safeguarding the heart from excessive vascular afterload. Na_V_ channels are essential elements of action potential generation and propagation in neurons and in skeletal and cardiac muscles. Na^+^ currents have also been measured in pulmonary and systemic vascular smooth muscle cells and systemic endothelial cells^4^. The functional role of Na^+^ channels in vascular cells is unclear but some studies have suggested that they may play a role in both regulation of Em and cell proliferation and apoptosis.

Channelopathies are an emerging group of disorders affecting ion channel function. Some of these disorders may be corrected by drugs and therefore, ion channels represent the second largest group of drug targets after G protein-coupled receptors^7^. Alterations in the K^+^ and Ca^2+^ channel density and activity at the cell surface strongly impact in the pulmonary circulation^4–6^. These diseases may result from a mutation in the encoding genes, from dysregulated gene expression, from the blockade of the ion channel protein by toxins or drugs or from other forms of impaired regulation of channel activity. Deleterious genetic variants in the KCNK3, KCNJ8, ABCC8 and ABCC9 genes (which encode the TASK1 and K_IR_6.1 channels and the auxiliary subunits SUR1 and SUR2, respectively) and possibly in KCNA5 (encodes for K_V_1.5 channels) have been associated to PAH, representing paradigmatic examples of channelopathies^8^. Downregulation of K^+^ channel expression is also a typical feature of the disease. Several K^+^ channels have been reported to be downregulated in animal models of PAH including K_V_1.1, K_V_1.5, K_V_1.6, K_V_2.1, K_V_3.1b, K_V_7.1, K_V_7.4 and TASK1 while others were upregulated such as K_V_11.1, K_Ca_1.1, K_Ca_3.1 and K_IR_6.1^9,10^. Some studies have also been carried out to analyze the alteration of specific cationic channels in patients with PAH. Notably, Yuan et al. demonstrated attenuated expression of Kv1.5 mRNA in lungs from PAH patients^9,10^ and reduced Kv1.1 and Kv4.3 channel mRNA have also been shown^4^. Downregulation of TASK1 expression and activity has also recently been demonstrated in PASMC from PAH patients^11^.

In the present study, the expression of the cationic channel encoding genes in lungs from patients with PAH was analyzed using a transcriptomic approach.

## Methods

Data was downloaded from the publically available Gene Expression Omnibus (GEO) dataset GSE113439 (https://www.ncbi.nlm.nih.gov/gds/). This dataset contained the gene expression profile of samples from explanted lungs of fifteen patients with pulmonary hypertension who underwent lung transplantation. Specimens were obtained from the peripheral area at the base of each organ. Lung samples obtained from the region of normal healthy tissue flanking lung cancer resections from eleven patients without pulmonary hypertension or left-side heart disease were used as controls. The PAH group (class 1 pulmonary hypertension) included six patients with idiopathic PAH (IPAH), four patients with PAH secondary to connective tissue disease (CTDPAH) and four patients with PAH secondary to congenital heart disease (CHDPAH). There was only one patient with chronic thromboembolic pulmonary hypertension (class 4 pulmonary hypertension) in the dataset which was not included for the present study. The sample preparation, RNA extraction, cDNA synthesis, microarray analysis, gene annotation, preprocessing and data curation and normalization followed standard procedures and are described in detail in the original paper and its supplement^12^. Briefly, microarrays were processed with Genechip® Human Gene Array and scanned on Affymetrix Genechip Scanners. The expression in the control and the PAH samples was compared for the whole transcriptome by the GEO2R analysis tool (https://www.ncbi.nlm.nih.gov/gds/) using the Benjamini & Hochberg approach for false discovery rate to calculate the adjusted p value. The principal component analysis was performed using the Past software (ver3.21, Oslo, Norway). The changes in channel expression between the different PAH subgroups (log[fold change] versus controls) were compared by regression analysis using Prism (Ver 7.04). P < 0.05 was considered statistically significant.

## Results and discussion

**Figure 1:**
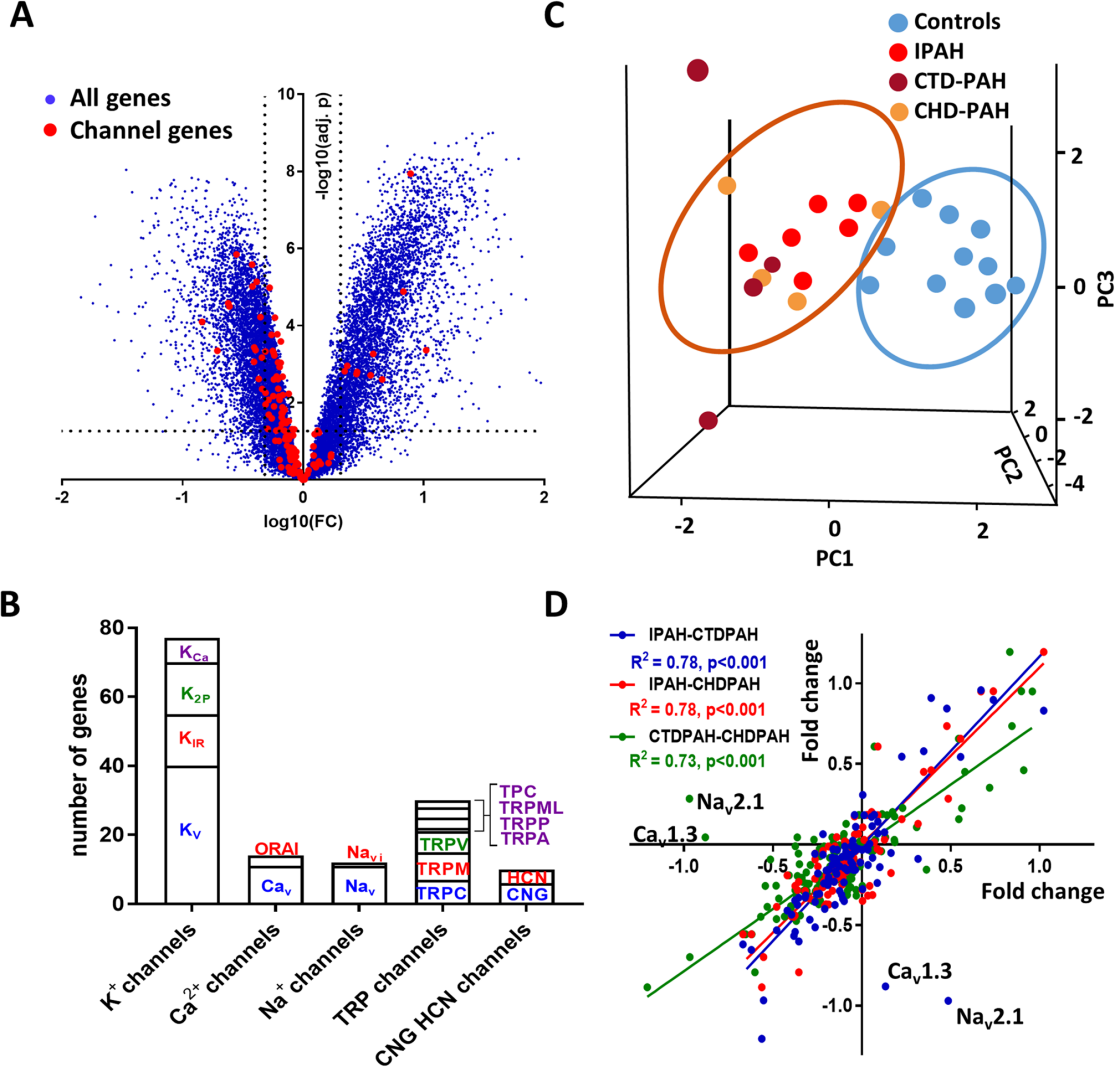
Channel gene expression profile analysis in pulmonary arterial hypertension. (**A**) Vulcano plot [log fold change against − log (adjusted p value)] showing the changes in mRNA transcripts for all identified genes (blue) and for cationic channel encoding genes (red). The vertical lines correspond to twofold up and down, respectively, and dots above the horizontal dotted line indicate adjusted *p* < 0.05. (**B**) Families and subfamilies of mRNAs encoding the pore forming alpha subunits of cationic channels identified in the control samples, (**C**) 3D principal component analysis performed with the 143 channel encoding mRNAs. (**D**) Regression analysis of the changes (fold change vs control) in the different subgroups of PAH: idiopathic PAH (IPAH), connective tissue disease associated PAH (CTD-PAH) and congenital heart disease PAH (CHD-PAH).

A total of 22,195 mRNA products were identified and 12,408 (56%) of them were significantly dysregulated in PAH compared to control lungs (adjusted p < 0.05). This large transcriptomic change is consistent with the advanced stage of the disease in these PAH patients undergoing transplant, which show complex lesions and accumulation of different PASMC and endothelial cells (PAECs), fibroblasts, myofibroblasts and inflammatory cells^2^. Therefore, the hypertensive lung transcriptome must reflect not only changes in the regulation of gene transcription in the different specific cell types but also changes in cell phenotypes and a different proportion of cell types within the lung. In fact, PAH cells show a hyperproliferative phenotype and share several characteristics with cancer cells^13^. The Vulcano plot shows an apparently symmetrical distribution of the changes in expression (blue dots in Fig. 1A) with 40.2% of these mRNA products significantly upregulated and 59.8% downregulated.

**Figure 2:**
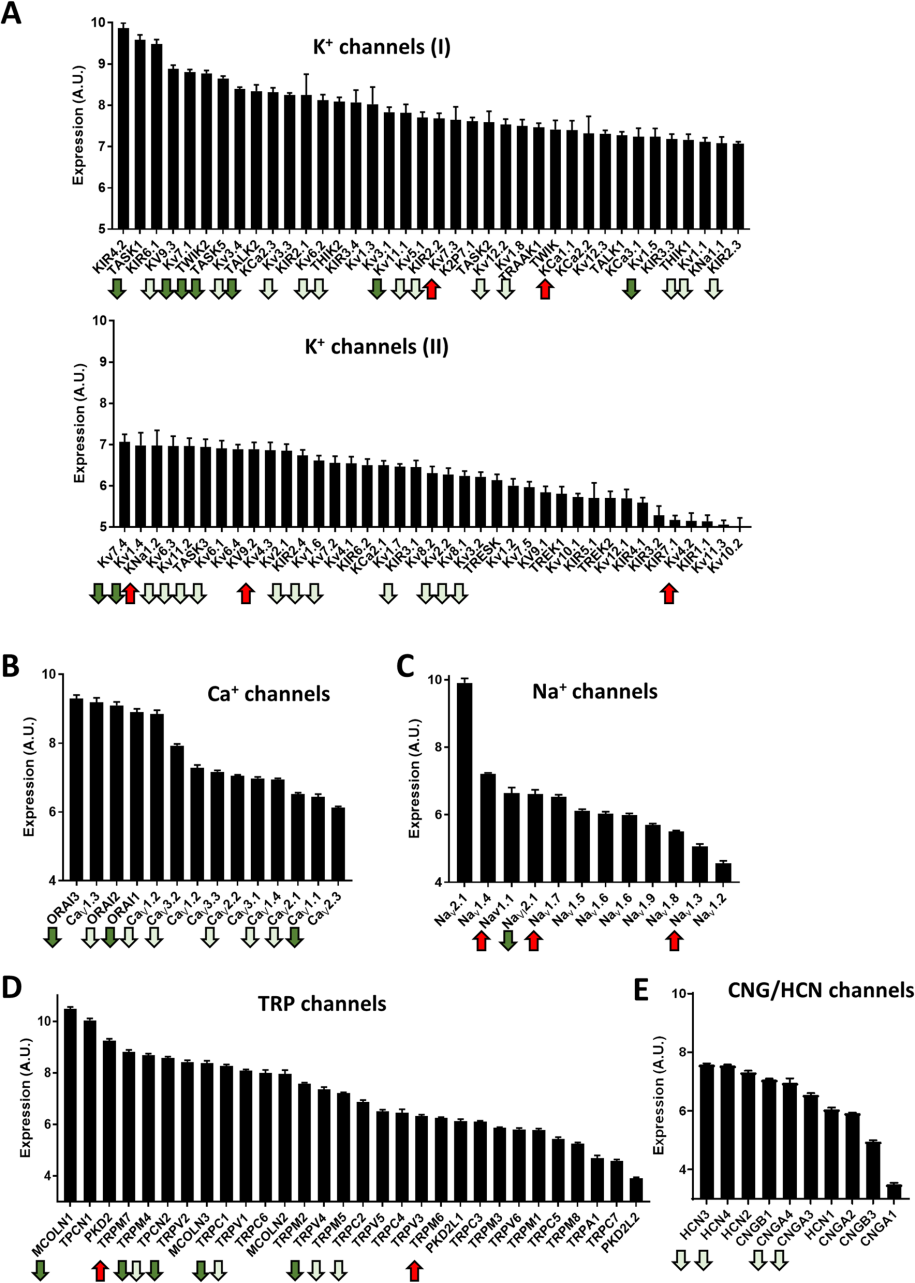
Relative abundance of ion channel encoding genes in the control lungs. Bars represent means ± SEM (n = 10). Arrows indicate significantly (adjusted *p* < 0.05) dysregulated channels in PAH: dark green for > twofold downregulation, light green for < twofold downregulation and red for > twofold upregulation.

**Figure 3:**
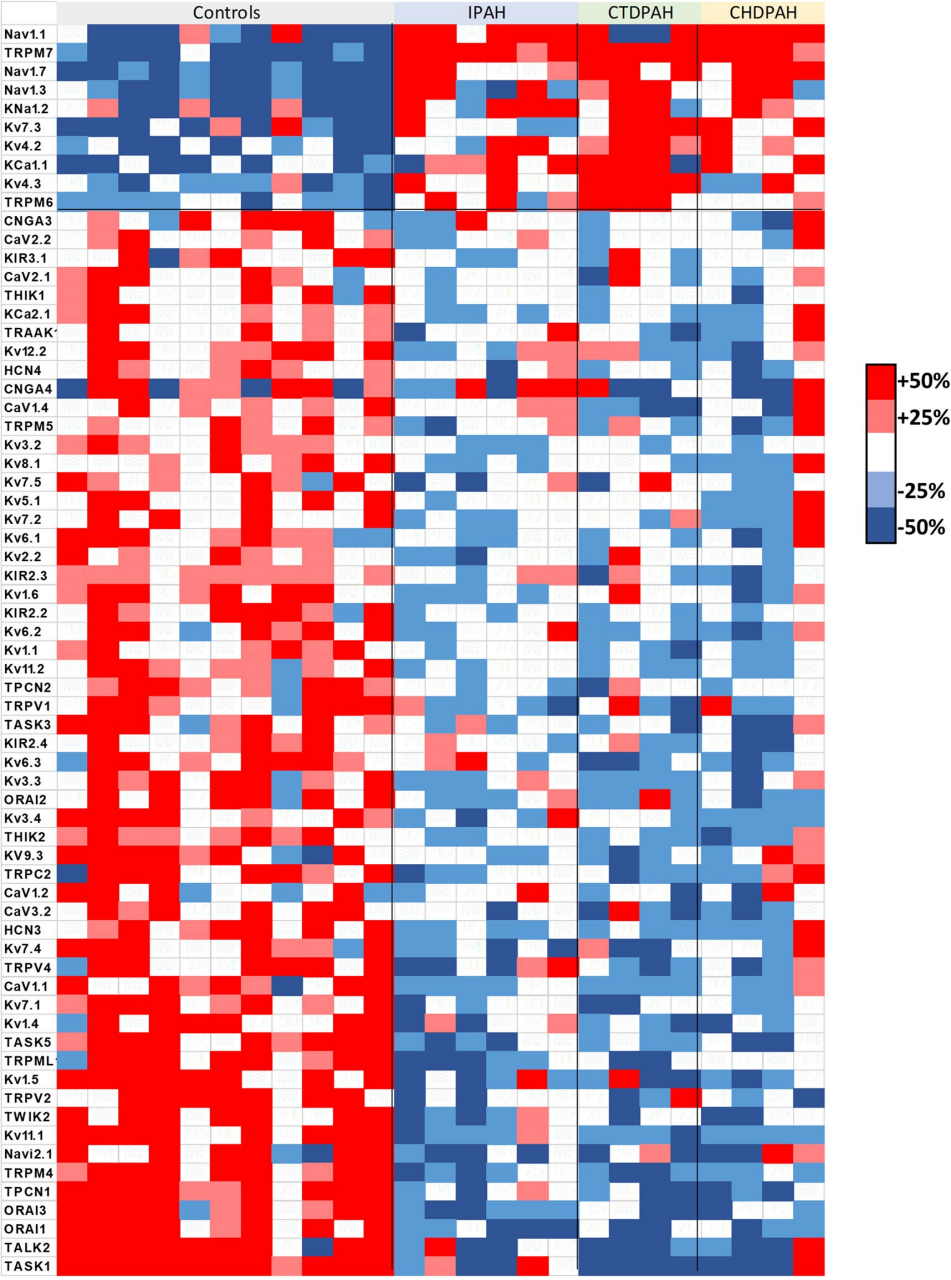
Heat map of genes encoding cationic channel that displayed significant difference (adjusted *p* < 0.05) in expression.

The different families and subfamilies of mRNAs encoding the pore forming alpha subunits of cationic channels identified are shown in Fig. 1B. K^+^ channels comprise the family of cationic channels with the highest relative abundance, followed by TRP channels; and to a lesser extent Ca^2+^, Na^+^ and CNG/HCN channels (Figs. 1B, 2). As expected, there were large differences in the relative expression of the different cationic channel genes in control lungs (Fig. 2), which gives a rough estimate of the importance of each channel. Among the 143 mRNA products identified as cationic channels, 66 of them (46.2%) were dysregulated in PAH with a strongly asymmetrical distribution in the Vulcano plot (red dots in Fig. 1A), with most of them downregulated (83.1%) and only 16.9% of them upregulated. This broad decrease in channel expression might be partly attributed to a relative loss or a phenotypic change in the electrically excitable cells in the lung in which voltage-gated cation channels are abundant, including smooth muscle, neurons and endocrine cells. The principal component analysis (PCA) for the whole transcriptome previously published^12^ indicated a complete separation between PAH subjects and normal controls. Herein, the unsupervised PCA applied to the 143 channel transcripts also clearly separated controls from PAH patients (Fig. 1C). This indicates that dysregulated cationic channel expression is a signature of the disease. However, the three different forms of PAH analyzed, i.e. IPAH, CTDPAH and CHDPAH, were indistinguishable in the PCA analysis. Two samples from the CTDPAH group were clearly separated from the rest of the PAH patients and further away from the controls. Moreover, the plots of the changes in channel gene expression in IPAH vs CTDPAH, IPAH vs CHDPAH and CTDPAH vs CHDPAH, shows an excellent correlation (Fig. 1D, P < 0.001 for the three pairs of comparisons). That confirms that the changes in channel gene expression were very similar in the three forms of PAH. The data was very internally consistent. The exceptions to this rule were only two genes, Ca_V_1.3 and Na_V_2.1, which were downregulated specifically in CTDPAH (supplementary Fig. 1). If confirmed, these might represent biomarkers of this specific subtype of PAH. The heat map for the genes encoding cationic channel that displayed significant difference (adjusted p < 0.05) in expression is shown in Fig. 3.

**Figure 4:**
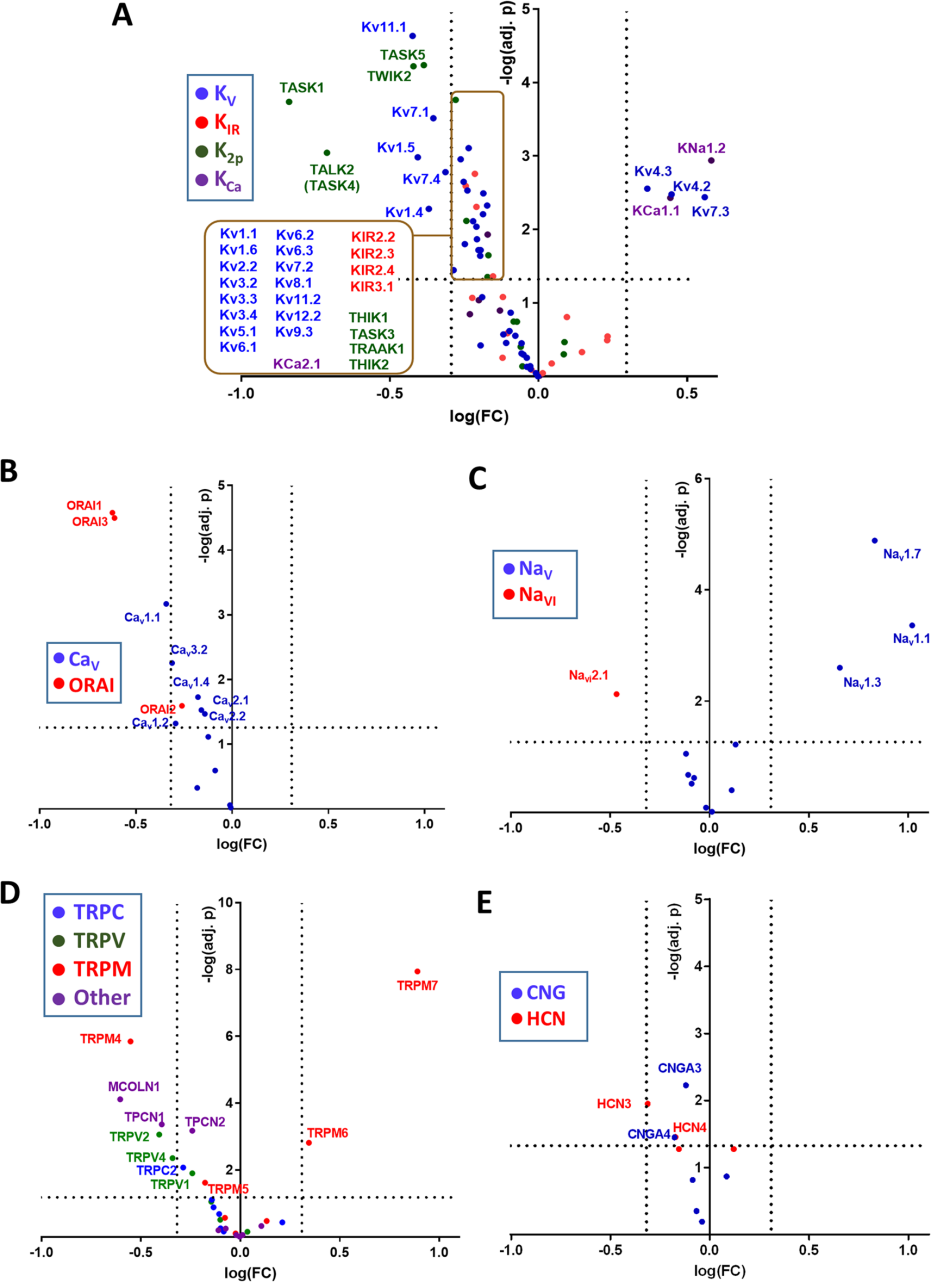
Vulcano plot [log fold change against − log (adjusted p value)] showing the changes in mRNA transcripts for cationic channel encoding genes (**A**) K^+^, B) Ca^2+^, C) Na^+^, D) TRP and E) CNG/HCN superfamilies. The vertical lines correspond to twofold up and down, respectively, and dots above the horizontal dotted line indicate adjusted *p* < 0.05.

The group of K^+^ channels is the largest family of cationic channels (Fig. 2A). The differential expression of K^+^ channels in PAH with 33 (44%) mRNAs encoding K^+^ channels significantly downregulated and 5 (7%) mRNAs upregulated is shown in Fig. 4A. Because K^+^ conductance is essential to maintain a normally polarized cell membrane, this reduced expression of K^+^ channels explains the depolarized Em observed in PASMC in PAH leading to increased contractility and cell proliferation^9^.

The analysis of the expression of each channel family (Fig. 4A–E) shows that the K^+^, Ca^2+^, TRP and CNG/HCN superfamilies of cationic channels present an asymmetrical distribution of their expression in PAH in the Vulcano plots with a majority of their members downregulated. The Na^+^ channel family with a smaller number of members shows a nearly symmetrical plot. The data with significant changes are shown in supplementary Figs. 2–5.

The K_V_ channel family contributed the largest number of dysregulated K^+^ channel genes (3 up- and 20 downregulated, Fig. 4A). Some of them have been previously reported to be downregulated in PAH including K_V_1.5 channels (KCNA5) which are key regulators of Em and are inhibited by vasoactive factors involved in PAH such as 5-HT, endothelin-1, thromboxane A_2_ and hypoxia^14–16^. In fact, K_V_1.5 downregulation is widely recognized as a hallmark of the disease in both humans and animal models^10,17,18^. Consistent with previous reports, other downregulated channels from this family are K_V_1.1, K_V_1.4 and K_V_1.6 channels. K_V_1.2 and K_V_2.1 channels, which were previously shown to be reduced in vitro by hypoxia in animal models of PAH^9,18^ were unchanged. However, the electrically-silent K_V_9.3 channels, which form a functional heteromer with K_V_2.1, were downregulated in the present and in the vitro study^18^. Several other K_V_ channels such as K_V_2.2, K_V_3.2, K_V_3.3, K_V_3.4, K_V_5.1, K_V_6.1, K_V_6.2, K_V_6.3, K_V_8.1 and K_V_12.2, that have been previously reported to be expressed in the pulmonary arteries^19^ but whose functional importance is unclear, were also modestly downregulated. In contrast, K_V_4.3 previously reported to be downregulated in vitro by hypoxia*,* as well as K_V_4.2, both of which generate transient currents, were found to be upregulated in PAH patients. On the other hand, the expression of K_V_11.1 channels, also known as hERG channels and involved in heart repolarization, was increased as measured by immunohistochemistry in the pulmonary arteries of humans affected by chronic obstructive pulmonary disease-associated pulmonary hypertension (class 3)^20^ but were significantly downregulated in the three forms of PAH in the present study.

In recent years, a key role of K_V_7 channels (KCNQ1-5) in the control of pulmonary vascular tone has been demonstrated^21–24^. K_V_7.1, K_V_7.3, K_V_7.4 and K_V_7.5 mRNAs are known to be expressed in rat pulmonary arteries^24,25^ but K_V_7.3 was not detected in vascular smooth muscle cells. Based on the effects of K_V_7 activators flupirtine and retigabine, these channels have been proposed as therapeutic targets for PAH^23,24^. In control human lungs, the five K_V_7 were expressed, with K_V_7.5 showing the lowest levels (Fig. 2A). Downregulation of K_V_7.1, K_V_7.4 and K_V_7.5 have been shown previously in animal models of PAH^21,23,24,26–28^. Herein K_V_7.1, K_V_7.2 and K_V_7.4 were downregulated in human PAH (Fig. 4A). Interestingly, K_V_7.3 channels were upregulated in the three forms of human PAH seemingly compensating for the loss of the other K_V_7 channels. This may also help to support the preserved Kv7 function in PAH^24^. Because K_V_7.3 are sensitive to flupirtine and retigabine, it would be worth analyzing the role of these channels in the context of PAH.

K_IR_ are expressed in vascular smooth muscle and endothelial cells^9,10^. The only change reported in PAH so far is an increase in K_IR_6.1, which forms the vascular K_ATP_ channel in the monocrotaline rat model of PAH^26^. The present results indicate that K_IR_6.1 was strongly expressed in the lungs (Fig. 2A) but unchanged by PAH and that most other K_IR_ are unchanged as well, except K_IR_2.2-K_IR_2.4 and K_IR_3.1 that were modestly downregulated (Fig. 4A).

K_Ca_ channels are activated by cytosolic Ca^2+^. The K_Ca_1.1 channel which is responsible of the large conductance K_Ca_ current (BKCa), was strongly upregulated in lungs from PAH patients (Fig. 4A). Similarly, these channels were upregulated in laser microdissected pulmonary arteries from IPAH patients^29^, suggesting the utility of BKCa openers for pharmacological interventions in PAH. On the contrary, K_Ca_2.1 which carries a small conductance Ca^2+^-activated K^+^ channel (SKCa) was modestly downregulated.

K_2P_ channels, also referred to as leak or background conductance channels, comprise a large family of voltage-independent K^+^ channels that control resting Em in smooth muscle cells. These K_2P_ channels are classified into six subfamilies according to their sequence and functional properties: the weak rectifying group (TWIK1, TWIK2, and K2P7.1), the mechano-gated group (TREK1, TREK2, and TRAAK), the acid-sensing group (TASK1, TASK3, and TASK5), the alkaline-sensitive group (TALK1, TALK2, and TASK2), the halothane-sensitive group (THIK1 and THIK2), and the spinal cord-expressed TRESK channel. Among this family, the mRNA of TASK1, TASK2, THIK1, TREK2 and TWIK2 are reported to be expressed in pulmonary arteries, playing a role in pulmonary vascular tone^30^. The present data also indicate a high expression of the mRNA of TALK2 (also named as TASK4), TASK5 and THIK2 in the whole lung (Fig. 2A). Remarkably, TASK1 has gained a reputation as an important player in PAH. In fact, loss of function mutations in *KCNK3* (TASK1) were the first channelopathy described in PAH^8,31^. Herein, the present results confirm the downregulation of TASK1 reported in both human and animal models of PAH^9–11^. In fact, TASK1 was the second most highly expressed K^+^ channel and the strongest downregulated channel in the channelome. Interestingly, other K_2P_ channels were also markedly downregulated, including TALK2, TWIK2 and TASK5. TALK2 heterodimerizes with TASK1^32^ and, thus, it might play a role together with TASK1 in controlling Em and tone in pulmonary arteries. Based on experiments carried in TWIK2 *knockout* mice, Pandit et al.^33^ suggested that these channels are involved in PAH. This concept was challenged by Lambert et al.^4^, which showed that mRNA expression of TWIK2 was unchanged in pulmonary arteries from IPAH patients. The present data, however, would argue in favor of the former possibility. The physiological role of TASK5 is unclear as it produces a weak current in transfected mammalian cell lines^34^. Other members of the family that were modestly downregulated include THIK1, THIK2, TRAAK1 and TASK3 and none of the channels of this family were upregulated.

Ca_V_ channels are activated by depolarization and the subsequent elevation in cytosolic Ca^2+^ constitutes the main mechanism for the activation of electrically excitable cells, including PASMC, and some non-excitable cells^35^. Pulmonary artery contraction depends on Ca^2+^-dependent pathways (mediated mainly by L-type Ca^2+^ channels) and Ca^2+^-independent pathways (mediated mainly by Rho kinase activation), and the relative role of each one may vary with age, the vasoconstrictor agent involved and the hypertensive state^36,37^. The downregulation of K^+^ channels in PAH as described above and the subsequent depolarization increases the activation of these voltage-gated Ca_V_ channels promoting contraction and proliferation. The Ca_V_1 subfamily (Ca_V_1.1–Ca_V_1.4) corresponds with the voltage-dependent channels known as L-type channels which are sensitive to classic Ca^2+^ channel blockers (verapamil, diltiazem and dihydropyridines such as nifedipine or amlodipine)^7^. These drugs constitute a first line therapy for PAH, but unfortunately only 10% of the patients respond to these drugs at clinically tolerable doses. Ca_V_1.2 and Ca_V_1.3 showed the highest expression in the lungs (Fig. 2B). Downregulation of Ca_V_1.2 has been previously reported in arteries from a model of persistent pulmonary hypertension of the newborn^38^. However, hypoxia upregulated these Ca_V_1.2 channels in mouse lungs^39^. The Vulcano plot shows that in PAH most Ca_V_ channels are modestly downregulated including the L-type Ca^2+^ channels Ca_V_1.1, Ca_V_1.2 and Ca_V_1.4 (Fig. 4B). The downregulation of Ca_V_1.x and the upregulation of Rho kinase in these patients^12^ might account for a higher role of Ca^2+^-independent pathways for pulmonary artery contraction^37^. It may also account for the low proportion of responders to L-type Ca^2+^ channel blockers. We also analyzed the relationship of the expression of the different Ca_V_1.x with the expression of K_V_ channels; the significant associations are shown in supplementary Fig. 6. It is interesting that in control patients there is an inverse association for certain K_V_ and the different Ca_V_1.x channels. Curiously, each K_V_ channel is only associated with a single Ca_V_1.x channel: K_V_1.3, K_V_4.2 and K_V_6.3 with Ca_V_1.1; K_V_12.1 with Ca_V_1.2; K_V_1.2, K_V_3.1, K_V_4.1, K_V_5.1 and K_V_6.2 with Ca_V_1.3 and finally K_V_4.3 with Ca_V_1.4 (black symbols in supplementary Fig. 6). On the contrary, in the lung from PAH patients these inverse associations were lost and all significant associations were direct (supplementary Fig. 6, blue symbols). The cause-effect relationships and their meaning are unclear. It is possible that K_V_ activity negatively regulates Ca_V_1.x expression and that this relationship is lost with the missing excitable phenotype in PAH cells. Ca_V_2.1, Ca_V_2.2 and Ca_V_2.3 correspond to the P/Q, N and R type, respectively, while the Ca_V_3 subfamily (Ca_V_3.1-Ca_V_3.3) correspond to the T-type channels. Hypoxia upregulated Ca_V_3.2 channels in mouse lungs^39^ while these channels were decreased in patients with PAH (Fig. 4B).

ORAI channels are store-operated Ca^2+^ entry channels which are activated by depletion of Ca^2+^ in the endoplasmic reticulum and do not share a common structure with voltage-gated like channels. It has been suggested that ORAI1 channels play a role in PAH^40^ but evidences are weak so far. The expression of ORAI2 was found increased in PASMC in culture from patients with iPAH^40^. ORAI2 was also upregulated in endothelial cells from PAH patients in a single cell transcriptomic analysis^41^. ORAI1 is involved in the transition of PASMC from a contractile to a proliferative phenotype^42^. However, the three ORAI genes were downregulated in the whole lungs from PAH patients in the present study (Fig. 4B) suggesting that its inhibition is not an appealing therapeutic strategy.

Na_V_ channels play a major role in regulating neuron excitability. Nonetheless, human pulmonary arteries expresses multiple Na_V._ channels^4,43^ although their functional role is unclear. The present study shows that the mRNA expression of Na_V_2.1 predominates in the lung (Fig. 2C). In lungs from PAH patients there was a strong upregulation of Na_V_1.1, Na_V_1.3 and Na_V_1.7 (Fig. 4C). In contrast, the sodium leak channel, Na_Vi_2.1, a voltage-insensitive member of the Na_V_ family, was downregulated in PAH lungs. The significance of these findings deserves further investigation.

The TRP channel family comprises seven subfamilies of cationic channels with diverse biophysical properties and physiological functions: the TRPC (“canonical”), the TRPV (“vanilloid”), the TRPM (“melastatin”), the TRPP (“polycystin”), the TRPML (“mucolipin”), the TRPA (“ankyrin”) and the TRPN (“nompC”, no mechanoreceptor potential C) families. TRP channels contribute to the modulation of cytosolic Ca^2+^ directly by carrying Ca^2+^ entry or indirectly by modulating the resting Em^4,44^. TRPC1, TRPC4 and TRPC6 are the major TRPC channels expressed in rat, mouse, and human pulmonary arteries^3,45^ and in the human lung (present results). TRPV channels, except TRPV2, are involved in thermic sensitivity. However, TRPV1, TRPV2, TRPV3 and TRPV4 mRNA were also detected in pulmonary arteries^44^ and in whole lung (present results). In contrast to other TRPs, TRPM4 and TRPM5 are impermeable to Ca^2+^ and its role in the lung is unknown. TRPM6 and TRPM7 are closely related; together they form a heterotetrameric channel permeable to divalent (Ca^2+^, Mg^2+^, Zn^2+^) and monovalent cations and play a role in Mg^2+^ homeostasis^44^. The mRNA of TRPM2, TRPM3, TRPM4, TRPM7 and TRPM8 were detected in pulmonary arteries^44^. Similarly, in the whole lungs analyzed in the present study, the highest expression was found in TRPM7, TRPM4, TRPM2 and TRMP5 channels. Among other TRP channels belonging to the TRPML, TRPP, TRPA and TPCN families, little is known about their expression and function in the lungs. The present study, however, shows a strong expression of the TRPP channel PKD2, the TRPML channels MCOLN1, MCOLN2, and MCOLN3 and the TPCN channels TPCN1 and TPCN2.

A number of changes were found in the literature about the expression of TRPs in pulmonary arteries of PAH patients^44^. TRPC1, TRPC4 and TRPC6 isoforms are upregulated in rat BMP2 knockdown PASMC, leading to increased Ca^2+^ entry and increased human PASMC proliferation and migration^46^. TRPC6 and TRPC3 mRNA and protein were also upregulated in lungs from IPAH patients^47^. Moreover, TRPC6 gene deletion suppresses chronic hypoxia-induced PH in mice^48^. All these findings strongly suggest a role for TRPC6 in PAH. However, the present results do not reproduce the upregulation of any TRPC mRNAs. The only significant change in this subfamily was a modest decrease in TRPC2 expression, probably irrelevant because TRPC2 is a pseudogene in humans. Both TRPV1 and TRPV4 were found to be upregulated in cultured PASMC from PAH patients^49^. In contrast, in the present PAH cohort there was a downregulation of lung TRPV1, TRPV2 and TRPV4. Interestingly, TRPV4 channels mediate a rise in endothelial intracellular Ca^2+^ concentration, leading to the activation of KCa3.1 (IKCa) and KCa2.3 (SKCa) currents and activating endothelial-dependent hyperpolarization (EDH) and relaxation in pulmonary arteries. The downregulation of TRPV4 is consistent with reduced EDH in pulmonary arteries from hypoxic mice^50^. In the TRPM family, TRPM7 was downregulated in cultured PASMCs from PAH patients and in rats with hypoxia-induced PAH and its inhibition exacerbates PAH^40^. Similarly, mice with a deletion of the kinase domain in TRPM7 channels in heterozygosis, show a severe systemic hypertensive phenotype induced by angiotensin II^51^. However, herein, TRPM7 and, to a minor extent, TRPM6 were upregulated in PAH. Conversely, TRPM5 was modestly reduced and TRPM4 was strongly downregulated. Other TRP channels downregulated include MCOLN1, TPCN1 and TPC2. Taken together, there was little correlation of the present findings regarding changes of TRP channel expression in PAH between the present data compared to isolated PASMC in animal models. This may reflect that TRP expression is quantitatively important in lung cells different from PASMC.

The CNG channels are permeable to Ca^2+^ and/or Na^+^ and are gated by cyclic nucleotides. They are involved in fluid absorption and CNGA2 has been reported to regulate Ca^2+^ in bovine PAECs^52^. CNGA3, CNGA4, CNGB2 and to a minor extent CNGA2 were expressed in the control lungs (Fig. 2E) and the CNGA3 and CNGA4 were downregulated in PAH lungs (Fig. 4E). HCN channels are permeable to Na^+^ and/or K^+^ and are activated by membrane hyperpolarization and by cyclic nucleotides^53^. They regulate neuronal excitability, pain and sinoatrial and atrioventricular node activities but their physiological role in the lung is unknown. Downregulation of the HCN4 channel in the atrioventricular node was observed in the monocrotaline rat model of PAH^54^. All isoforms of HCN (Fig. 2E) were present in the lung and HCN3 and HCN4 were moderately downregulated in PAH lungs (Fig. 4E).

A limitation of this study is that control samples were from neoplasia-free areas of lungs from patients with lung cancer, which might obviously affect the results. K^+^ channels, including Kv1.5, have been implicated in the abnormal growth of lung cancer cells^55^. K_V_1.5 expression negatively correlated with tumor grade; the higher the histologic tumor grade the lower the K_V_1.5 expression. It is unknown whether healthy cells from cancer patients also show dysregulated K^+^ channels. However, if anything these issues should hide the differences observed herein. Moreover, PAH samples were obtained from advanced-stage, severe ill patients undergoing transplantation. Then, some changes found may be the consequence of a critical health condition rather than a pathophysiological causative factor of the disease. An additional important limitation of the present study is that mRNA expression is not always translated into protein and even when protein is present it might not traffic to the membrane or may not form a functional channel. However, the study has the strength of using clinically relevant samples in contrast with much of the previous work done on cell culture from PAH patients or controls with or without exposure to hypoxia, which can be problematic because channel expression can be altered by culture passage. Some studies have analyzed specific channels genes in PASMC or PAECS or in pulmonary arteries. These include cells from manually dissected medium and small pulmonary arteries, illustrative of vascular changes but which may not be very representative of the complex lesions present in the microcirculation in PAH lungs, such as onion-skin lesions, plexiform core lesions and dilation lesions^2^.

In conclusion, the dysregulation of cationic channel expression is a signature of PAH with multiple channels being downregulated and a few of them upregulated. Changes were very similar in IPAH, CTDPAH and CHDPAH. When compared to changes reported in the literature, this analysis of the cationic channel transcriptome in PAH: 1) is in agreement with previously published changes in the expression of channels which play an important and widely recognized pathophysiological role in the disease such as the downregulation of TASK1 and K_V_1.5 in humans and animal models; 2) supports previous preliminary reports pointing to the dysregulation of several K^+^ channels including the downregulation of K_V_1.1, K_V_1.4, K_V_1.6, K_V_7.1, K_V_7.4, K_V_9.3 and TWIK2 and the upregulation of K_Ca_1.1; 3) argues against the proposed role of other K^+^ channels including K_V_1.2, K_V_2.1, K_V_4.2, K_V_4.3, K_V_11.1, K_IR_6.1 and ORAIs and 4) points to other novel cationic channels dysregulated such as Kv7.3, TALK2, Ca_V_1 and TRPV4 which might play a pathophysiological role in PAH. The significance of other changes found in Na^+^ and other TRPs remains to be investigated.

## Supplementary Information

Supplementary Information.

## Data Availability

The data that support the findings of this study are available in Gene Expression Omnibus (GEO) at https://www.ncbi.nlm.nih.gov/gds/, reference number GSE113439.
