# Supplementary material for: Transcriptomic profile of cationic channels in human pulmonary arterial hypertension
Source: Sci Rep. 2021 Aug 4;11:15829. doi: 10.1038/s41598-021-95196-z (PMC8338963; doi:10.1038/s41598-021-95196-z)
Supplement: Supplementary file 1 — Supplementary Information. [file 41598_2021_95196_MOESM1_ESM.pdf]

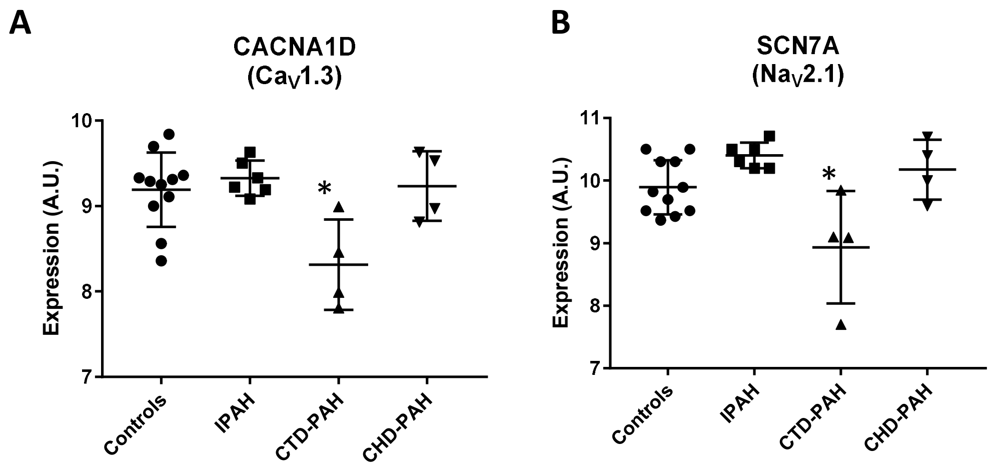

Supplementary figure 1. mRNA expression of CACNA1D and SCN7A genes in controls and patients with idiopathic PAH (IPAH), congestive tissue disease associated PAH (CTD-PAH) and congenital heart disease PAH (CHD-PAH). Results are shown as scatter plot and lines indicate means and SD. P <0.05 vs iPAH and CHD-PAH.

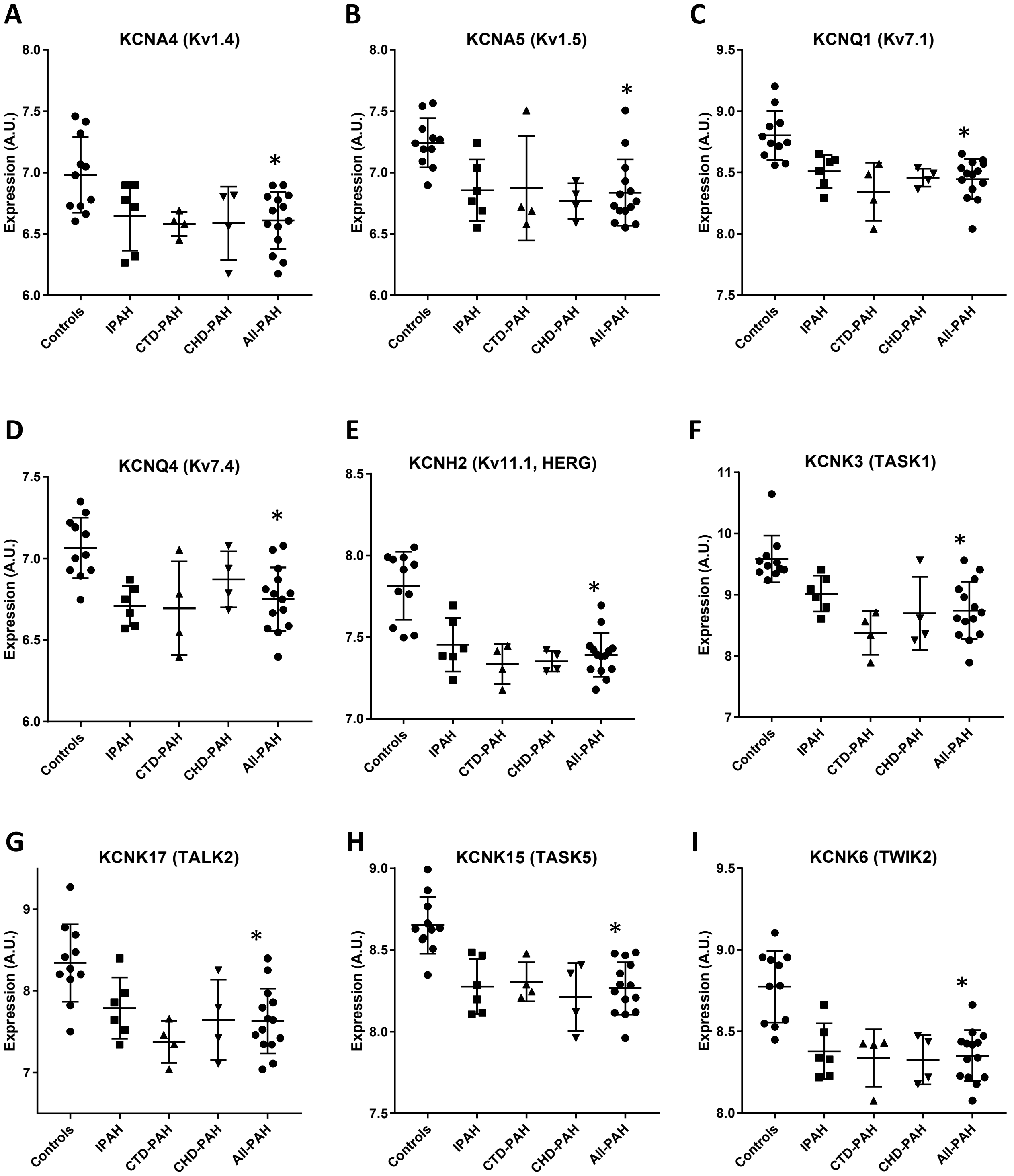

Supplementary figure 2. mRNA expression of K+ channel genes with a large (> 2 fold) and significant (adjusted p < 0.05) downregulation in controls and patients with idiopathic PAH (IPAH), congestive tissue disease associated PAH (CTD-PAH) and congenital heart disease PAH (CHD-PAH). Results are shown as scatter plot and lines indicate means and SD. * Adjusted p < 0.05.

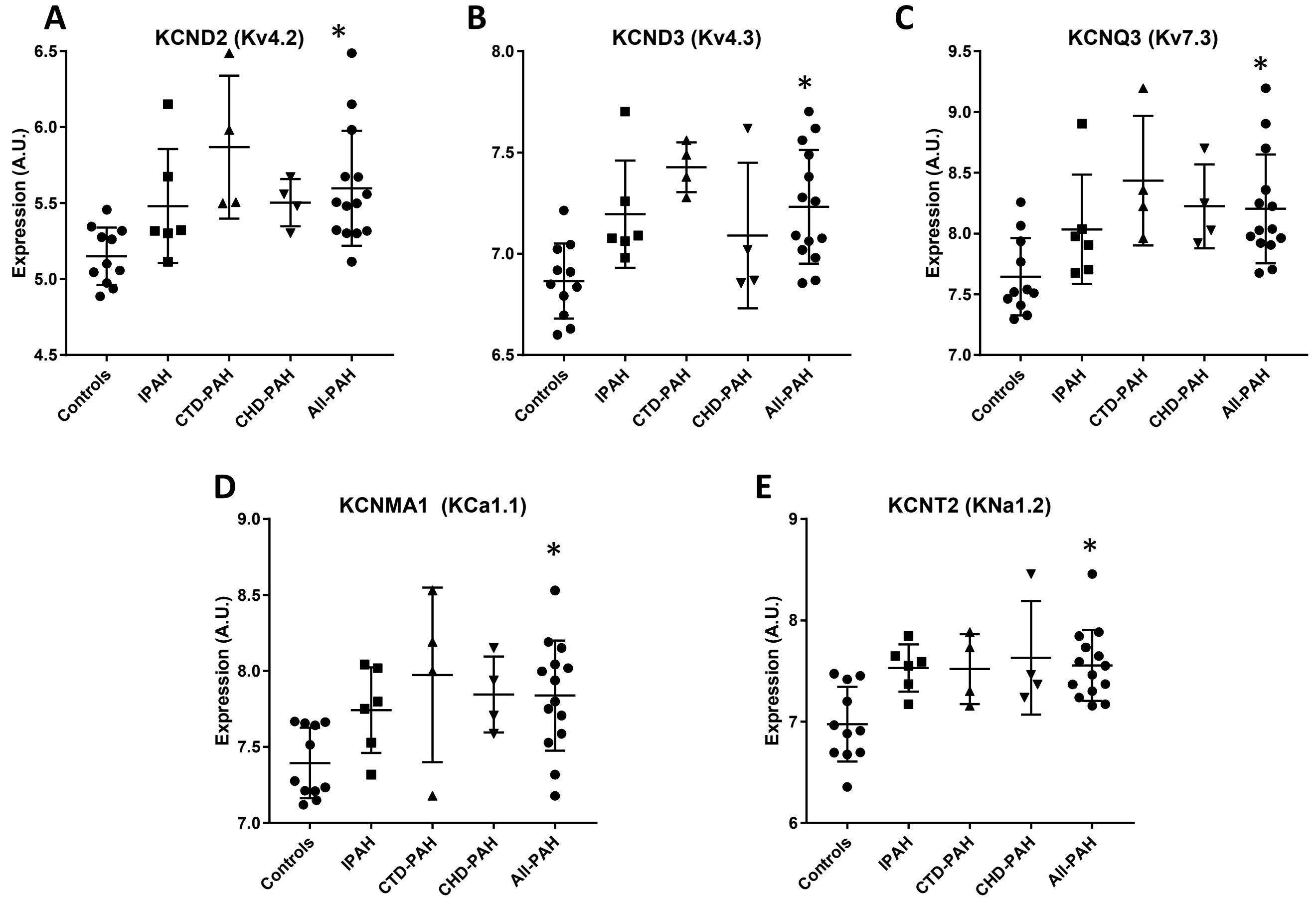

Supplementary figure 3. mRNA expression of K^+ channel genes with a large (> 2 fold) and significant (adjusted $p < 0.05$) upregulation in controls and patients with idiopathic PAH (IPAH), congestive tissue disease associated PAH (CTD-PAH) and congenital heart disease PAH (CHD-PAH). Results are shown as scatter plot and lines indicate means and SD. * Adjusted $p < 0.05$.

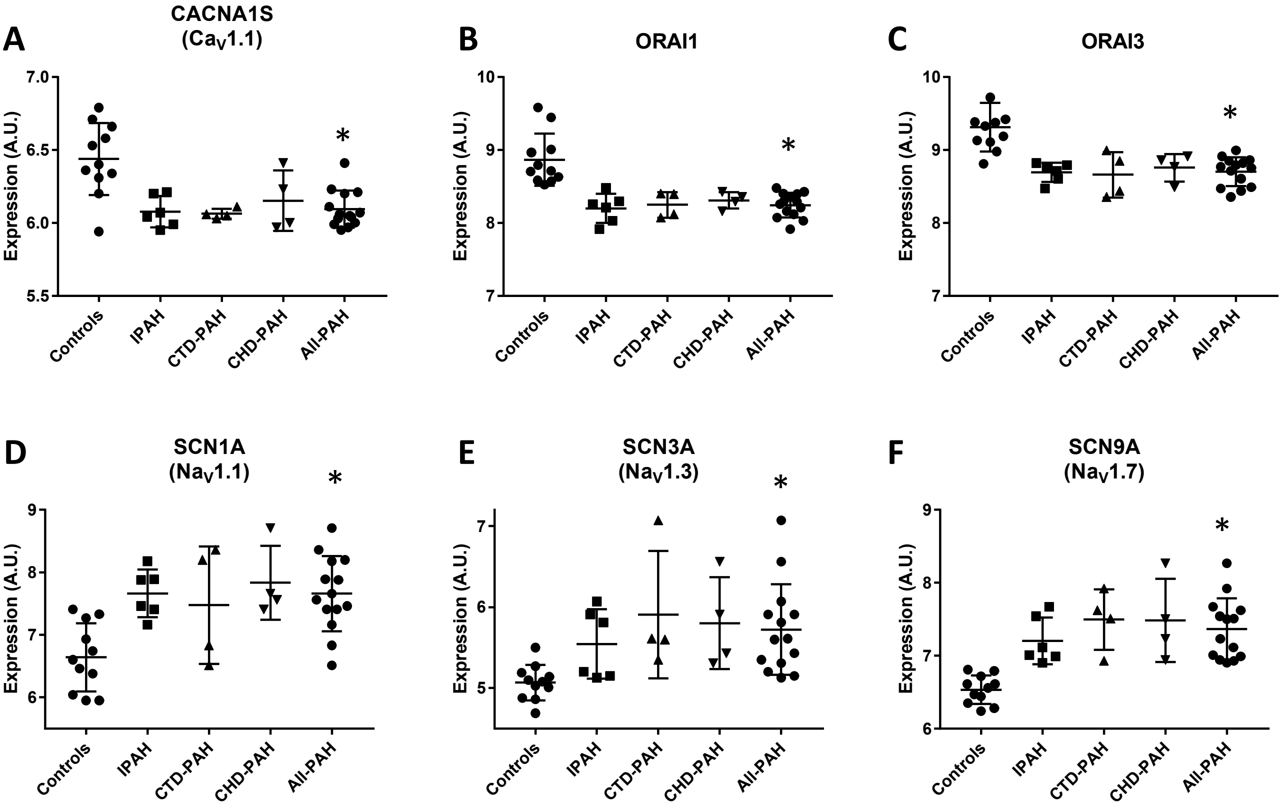

Supplementary figure 4. mRNA expression of Ca^{2+} and Na^{+} channel genes with a large (> 2 fold) and significant (adjusted $p < 0.05$) change in controls and patients with idiopathic PAH (IPAH), congestive tissue disease associated PAH (CTD-PAH) and congenital heart disease PAH (CHD-PAH). Results are shown as scatter plot and lines indicate means and SD. * Adjusted $p < 0.05$.

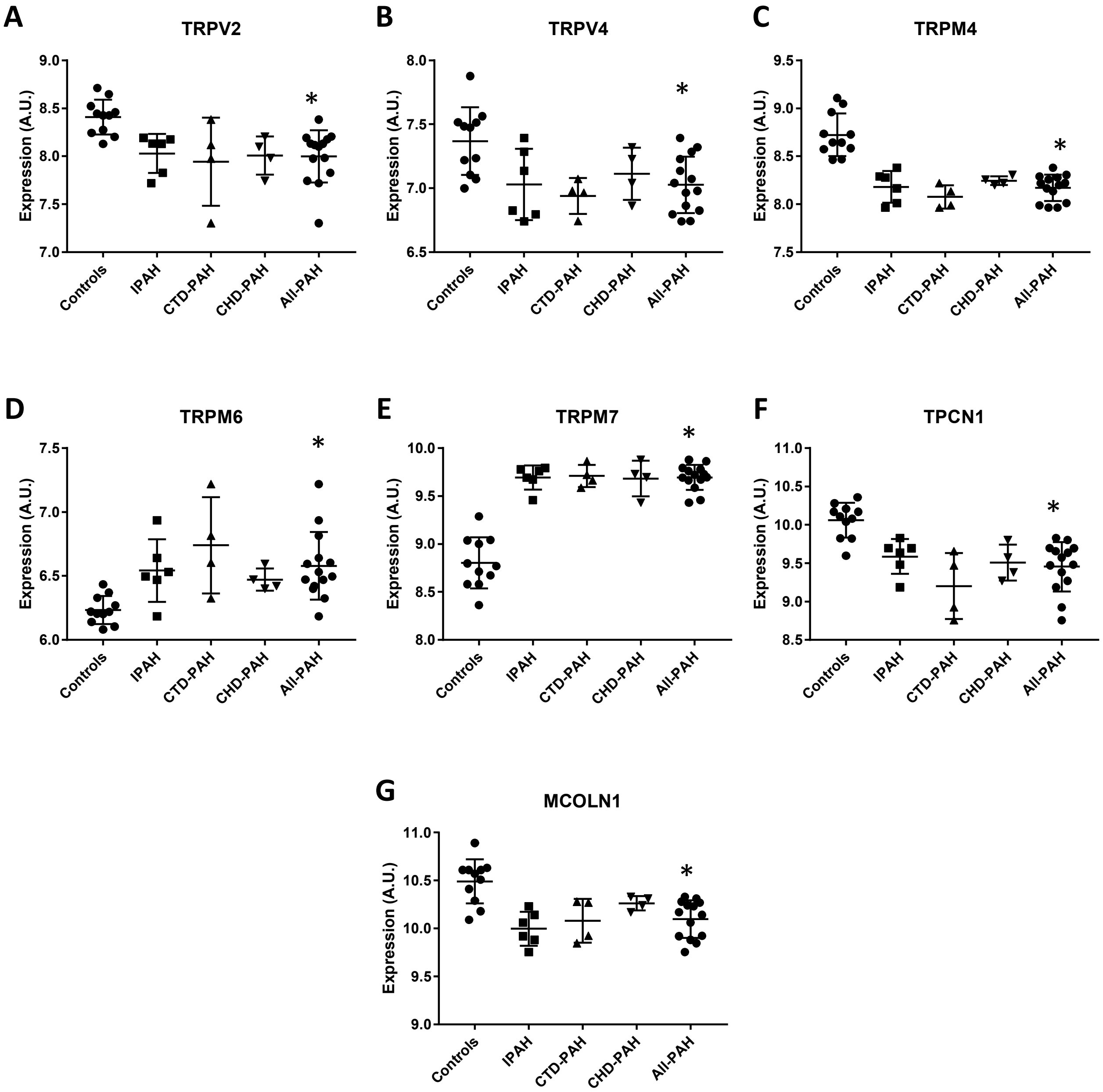

Supplementary figure 5. mRNA expression of TRP channels genes with a large (> 2 fold) and significant (adjusted p < 0.05) change in controls and patients with idiopathic PAH (IPAH), congestive tissue disease associated PAH (CTD-PAH) and congenital heart disease PAH (CHD-PAH). Results are shown as scatter plot and lines indicate means and SD. * Adjusted p < 0.05.

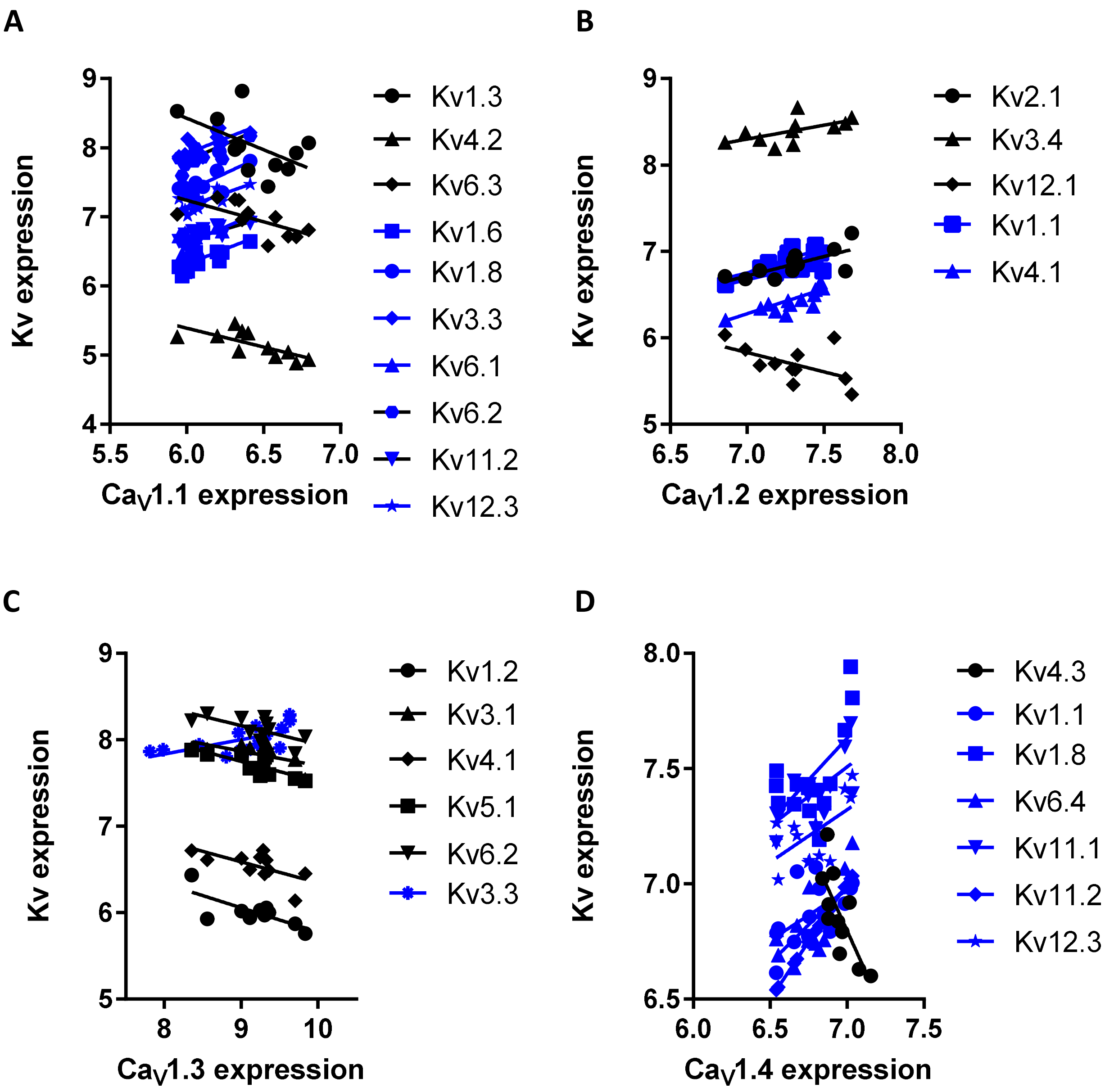

Supplementary figure 6. Significant correlations of mRNA expression of Ca$_V$1.X channels with Kv channels in controls (black symbols) and PAH patients (blue symbols).
